# Base Excision Repair AP-Endonucleases-Like Genes Modulate DNA Damage Response and Virulence of the Human Pathogen *Cryptococcus neoformans*

**DOI:** 10.3390/jof7020133

**Published:** 2021-02-12

**Authors:** Rayssa Karla de Medeiros Oliveira, Fabián Andrés Hurtado, Pedro Henrique Gomes, Luiza Lassi Puglia, Fernanda Fonsêca Ferreira, Kunal Ranjan, Patrícia Albuquerque, Márcio José Poças-Fonseca, Ildinete Silva-Pereira, Larissa Fernandes

**Affiliations:** 1 Department of Cell Biology, Institute of Biological Sciences, University of Brasília, Brasília 70.910-900, Brazil; oliveirarkm@gmail.com (R.K.d.M.O.); fahejml@gmail.com (F.A.H.); pedro.hqgomes@gmail.com (P.H.G.); luiza_puglia@hotmail.com (L.L.P.); 2 Department of Genetics and Morphology, Institute of Biological Sciences, University of Brasília, Brasília 70.910-900, Brazil; fonseca.fernandaf@gmail.com (F.F.F.); kukkukr.ranjan@gmail.com (K.R.); 3 Faculty of Ceilândia, University of Brasília, Brasília 72.220-275, Brazil; palbuquerque@unb.br

**Keywords:** *Cryptococcus neoformans*, base excision repair, AP-endonucleases-like genes, *APN1* and *APN2*, virulence

## Abstract

Pathogenic microbes are exposed to a number of potential DNA-damaging stimuli during interaction with the host immune system. Microbial survival in this situation depends on a fine balance between the maintenance of DNA integrity and the adaptability provided by mutations. In this study, we investigated the association of the DNA repair response with the virulence of *Cryptococcus neoformans*, a basidiomycete that causes life-threatening meningoencephalitis in immunocompromised individuals. We focused on the characterization of *C. neoformans* *APN1* and *APN2* putative genes, aiming to evaluate a possible role of the predicted Apurinic/apyrimidinic (AP) endonucleases 1 and 2 of the base excision repair (BER) pathway on *C. neoformans* response to stress conditions and virulence. Our results demonstrated the involvement of the putative AP-endonucleases Apn1 and Apn2 in the cellular response to DNA damage induced by alkylation and by UV radiation, in melanin production, in tolerance to drugs and in virulence of *C. neoformans* in vivo. We also pointed out the potential use of DNA repair inhibitor methoxy-amine in combination with conventional antifungal drugs, for the development of new therapeutic approaches against this human fungal pathogen. This work provides new information about the DNA damage response of the highly important pathogenic fungus *C. neoformans*.

## 1. Introduction

DNA integrity and genome stability are crucial for the adaptation and evolution of the species [1,2,3]. The ability of a pathogenic microorganism to survive in distinct environments, under different types of stresses, is usually related to phenotype plasticity. However, extreme alterations in the genome can compromise an organism’s viability, so cells employ different mechanisms to preserve the genome from the mutagenic action of genotoxic agents and to ensure the correct chromosome duplication and transmission to the next generations. Molecular processes underlying genome stability include DNA damage sensors, DNA repair pathways and cell cycle checkpoints [2,4,5].

DNA repair is mediated by several proteins, in a complex system of signal cascades, DNA damage sensors and effector enzymes [4,6,7]. The base excision repair (BER) pathway is responsible for the recognition and correction of non-bulky DNA lesions such as oxidized bases and abasic sites (AP-sites) [8,9]. The BER pathway is essential to maintain cell integrity and the organism’s survival under oxidative stress.

The BER pathway involves five steps to remove the DNA lesion and to restore DNA integrity: (i) excision of a damaged or inappropriate base by glycosylase proteins; (ii) incision of the phosphodiester backbone at the resulting abasic (AP) site by AP-endonucleases (APE); (iii) termini clean-up to permit unabated repair synthesis and/or nick ligation; (iv) gap-filling to replace the excised nucleotide by DNA polymerase; and (v) sealing of the remaining DNA nick by ligase [8,10]. In mammals, the disruption of APE proteins blocks the entire BER pathway [11].

Two major classes of AP-endonucleases have been characterized in *Escherichia coli*. The Xth family (exonuclease III) is constitutive and abundant, representing about 90% of the total AP activity. The Nfo (endonuclease IV) family represents 10–50% of the AP-endonuclease activity in the cells [12,13,14]. AP-endonucleases may act in functions beyond the AP-site cleavage. The most extensively studied AP-endonuclease proteins are human APE1 and APE2. APE1 possesses multifunctional activity, acting as a transcription factor and a redox activator [15]. Recently, it was demonstrated that APE1 and APE2 are involved in epigenetic and chromosomal alterations, facilitating the recruitment of the transcriptional machinery to specific promoters and enabling the formation of checkpoint protein complexes [16,17,18].

In *Saccharomyces cerevisiae* Apn1 is the major AP-endonuclease and belongs to the *E. coli* Nfo family. *S. cerevisiae* Apn2 shares homology with *E. coli* Xth and human APE1 and APE2 and has a back-up role in the repair of AP sites when Apn1 is absent [12,19]. Both possess APE and DNA 3′-phosphodiesterase activities [20]. Unlike *apn1*Δ mutants, *S. cerevisiae apn2*Δ mutants exhibit normal sensitivity to the alkylating agent methyl methane-sulfonate (MMS) and show no increase in spontaneous mutation rates, suggesting a less prominent role in the repair of AP sites [12,20,21]. Studies with *Candida albicans* revealed that Apn1 has minor participation in the susceptibility to DNA-damaging agents and drug response [22]. The function of *C*. *albicans* Apn1 is still unclear. In *Schizosaccharomyces pombe*, differently from *S. cerevisiae*, the Apn2 protein resolves approximately 90% of the abasic lesions on the BER pathway [23]. *S. pombe* Apn2 is phylogenetically closer to human APE proteins and possesses important domains involved in protein–protein interactions [23,24].

The mechanisms involved in the cellular response to DNA damage and repair are of great clinical significance [25,26,27,28,29]. DNA damage-inducing drugs, such as cisplatin and temolozomide and DNA repair inhibitor drugs, such as methoxy-amine, are used in cancer therapy [30,31,32]. Several antibiotic drugs are known to cause chemical damage to DNA. Drugs from the bleomycin family bind to DNA and induce double-strand breaks [33]. Antifungal drugs, like polyenes, induce the production of reactive oxygen species (ROS) [34]. Antimicrobial-induced ROS, such as hydroxyl radicals, damage the DNA through the formation of abasic sites, DNA strand breaks, and the incorporation of oxidized guanine residues into the genome [33,35].

DNA damage and repair are also involved in microbial virulence. Mutations are involved in the microevolution of several pathogens, while pathogenic microbes have developed several mechanisms to prevent DNA damage, which can be induced, for example, by reactive oxygen, nitrogen, and chloride species during host response to infection [36,37,38,39]. *C. neoformans*, the etiological agent of cryptococcosis, is the major cause of illness in people living with HIV/AIDS worldwide [40,41]. This ubiquitous basidiomycete is highly adapted to extreme environmental conditions such as desiccation, high temperatures and UV exposure [41,42]. Considering both environmental and host-related processes that might induce DNA damage in *C. neoformans* cells, we decided to investigate the involvement of the BER pathway in stress resistance and virulence in this fungal pathogen by the functional characterization of the BER pathway putative genes encoding Ap-endonucleases 1 and 2.

## 2. Materials and Methods

### 2.1. Strain Maintenance

The *C. neoformans* serotype A strain H99 (kindly donated by Prof. J. Andrew Alspaugh, Duke University, Durham, NC, USA) was used to generate *apn1*∆, *apn2*∆, the double *apn1*∆*apn2*∆ mutants and the complemented strains. All the strains were stored in 35% glycerol at −80 °C. Yeasts were cultured on YPD (Yeast extract – Peptone – Dextrose) agar plates (1% yeast extract, 2% peptone, 2% dextrose and 1.5% agar pH 5.6) and incubated at 30 °C, unless stated otherwise. Single colonies were inoculated in liquid YPD and grown overnight at 30 °C, under agitation (150 rpm). Before the experiments, cells were centrifuged at 4000 rpm and washed in phosphate-buffered saline (PBS) 1× to remove the culture medium.

### 2.2. Phylogenetic Analysis of the Predicted C. neoformans Apn1 and Apn2 Proteins

The amino acid sequences of previously identified AP- endonucleases of *S. cerevisiae* (ASP85051.1), *S. pombe* (NP_595522.1), *C. albicans* (KHC89247.1), and *Homo sapiens* (AAD43041.1) were obtained from the Broad Institute Fungal Genome Initiative (https://www.broadinstitute.org/fungal-genome-initiative, accessed on 11 February 2021), FungiDB (http://fungidb.org/fungidb/, accessed on 11 February 2021) and the NCBI (National Center for Biotechnology Information) data base (https://www.ncbi.nlm.nih.gov/gene, accessed on 11 February 2021). All of the sites were accessed on 5 November 2014. These sequences were compared with the genome of *C. neoformans* var *grubii* H99 by using the Blastp tool (https://blast.ncbi.nlm.nih.gov/Blast.cgi?PAGE=Proteins, accessed on 11 February 2021). Multiple sequence alignments were performed by MUSCLE 3.7 (Multiple Sequence Comparison by Log expectation): http://phylogeny.lirmm.fr/phylo_cgi/simple_phylogeny.cgi (accessed on 11 February 2021) Task_type  =  muscle. From the comparison with the *S. cerevisiae*, *C. albicans* and *S. pombe*, CNAG_05468 and CNAG_04268 were assumed to correspond to the *C. neoformans* AP endonuclease 1 (Apn1) and Apn exodeoxyribonuclease III (Apn2) putative coding sequences, respectively.

### 2.3. Disruption of the APN1- and APN2-Like Genes

The *C. neoformans APN1* and *APN2* putative genes were deleted from the serotype A H99 strain background by biolistic transformation and homologous recombination of the deletion cassette. The double-joint PCR (DJ-PCR) strategy [43] was used to construct the deletion cassettes. The first round of the DJ-PCR method was performed with primer pairs to amplify the 5′- and 3′-flanking regions of the target genes, using the H99 strain genomic DNA as a template. The dominant selection markers for Nourseotricin (NATr) and Hygromycin B (HYGr) were amplified with the M13Fe (M13 forward extended) and M13Re (M13 reverse extended) primers from the pPZPNAT and pPZPHYG73 plasmids, respectively. In the second round of PCR, the first-round amplicons were used as a template for the amplification of the target gene disruption cassettes with the 5′ or 3′ region of the NAT-split or HYG-split marker. The split gene disruption cassettes were introduced into the H99 strain by using the biolistic transformation method [43]. Stable Nourseothricin- or Hygromycin B- resistant transformants were screened by colony PCR with the primer sets listed in Table S1. The genotypes of all the positive transformants were verified by PCR analysis (Figure S1) and described in Table S2.

### 2.4. Phenotype Assays

For resistance to stress conditions, serial dilutions of cell suspensions (10^6^ to 10^2^ cells) were plated on YPD agar supplemented with 0.03 mM menadione (MND), 4 mg/mL sodium nitrite (NaNO_2_), 1 or 3 mM hydrogen peroxide (H_2_O_2_), 1.5 M sodium chloride (NaCl), 1.5 M potassium chloride (KCl), 32.4 mg/mL calcium sulfate (CaSO_4_), 110 µg/mL hydroxyurea (HU), 0.03% ethyl methane-sulfonate (EMS), 2.5 M sorbitol, 0.03% methyl methane-sulfonate (MMS), and 0.5 or 1 mg/mL caffeine. Plates were incubated at 30 or 37 °C for 48 h. For the capsule expansion measurement, cells were inoculated into chemically defined minimal medium (15 mM dextrose, 10 mM MgSO_4_, 29.4 mM KH_2_PO_4_, 13 mM glycine, 3 μM thiamine (pH 5.5)), incubated at 30 °C, at 150 rpm, for 2 days and counterstained with India ink. The diameter of each cell body and surrounding capsule was measured by ImageJ (Fiji) software. The average diameter of the capsule was calculated by subtracting the cell body diameter from the whole cell diameter (cell body + capsule). At least 100 cells were measured for each assay.

For the analyses of phospholipase or urease activities, 10^6^ cells were spotted on Agar Egg emulsion medium or on Christensen’s urea agar (0.1% peptone, 0.5% NaCl, 0.2% KH_2_PO_4_, 0.1% glucose, 2% urea, 0.0016% phenol red), respectively, and incubated for 72 h at 30 °C. Phospholipase activity (Pz) was measured by the ratio of the colony diameter (DC) to the colony diameter plus precipitation zone (DCP). Pz = 1 = no activity; 1 > Pz > 0.63 = phospholipase activity [44]. Urease activity was analyzed by the change from yellow to pink of Christensen’s urea medium color.

For the melanin production assay, cells were inoculated in liquid minimal medium supplemented with 1 mM l-DOPA (l-3,4-dihydroxyphenylalanine) (Sigma-Aldrich, St. Louis, MO, USA) and incubated at 30 or 37 °C, with shaking at 150 rpm, for 4 days in dark. All the assays were performed as three independent experiments.

### 2.5. Growth Curve Analysis

Cell suspensions were adjusted to the concentration of 1 × 10^5^ /mL in YPD medium. In a 96-well polystyrene microplate, 1 × 10^4^ cells/well were inoculated and the plates were incubated under continuous agitation at 30 or 37 °C. Optical density was measured every 30 min at 600 nm by the Eon biotek spectrophotometer (BioTek Eon Microplate Spectrophotometers, Winooski, VT, United States) for 96 h. All the assays were performed as three independent experiments.

### 2.6. Evaluation of the Yeast Cells’ Resistance to Genotoxic Stress

For the UV irradiation susceptibility analysis, serial dilutions of cell suspensions were spotted on YPD agar plates and exposed to UV irradiation of 60 to 480 J/m^2^ at 254 nm in a UV cross-linker chamber UVP CX-2000 (Fisher Scientific, Leicestershire, UK). The plates were incubated at 30 °C for 2 days and photo-documented. For viability analyses, 5 × 10^2^ cells were washed, plated on YPD plates and exposed to 120, 240 or 480 J/m^2^ at 254 nm in a UV cross-linker chamber. Plates were protected from light and incubated at 30 °C for 2 days for colony-forming unit (CFU) counting. Yeast cells were spotted onto YPD agar containing the DNA damage stress-inducing agents: HU (110 mM), MMS (0.03%), EMS (0.04%), *N*-ethyl-N-nitrosourea (ENU) (200 µg/mL), ethidium bromide solution (EtBr) (20 µg/mL).

For cell viability analyses, yeasts were inoculated in liquid YPD containing MMS (0.1% or 0.2%), EMS (0.1% or 0.2%), methoxy-amine (MX) (0.417 mg/mL to 1.67 mg/mL), or Zeocin (0.4% to 0.16%) and incubated for 1 h, at 30 or 37 °C, at 200 rpm. Five hundred cells were washed, plated on YPD plates and incubated at 30 °C for 2 days for CFU counting. The assay was performed as three independent experiments. Statistical test One-way ANOVA with Dunnett’s post-test was applied, to compare the results.

For the evaluation of DNA fragmentation, 5 × 10^7^ cells of the *C. neoformans* strains were exposed to 0.08% Zeocin for 1 h in YPD liquid medium at 30 °C at 200 rpm. Cells were washed with PBS, and DNA was extracted using the Smash and Grab protocol. One µg of genomic DNA was loaded in 1.5% agarose gel stained with 0.5 µg/mL EtBr. Genomic DNA from the unexposed H99 strain was used as control. The pattern of DNA migration on agarose gel was compared to genomic DNA of unexposed H99 to define the presence of DNA trail and fragmentation. The assay was performed as three independent experiments.

For the evaluation of the combined action of MX and H_2_O_2_, 1 × 10^6^ cells/mL were cultured in YPD medium containing 0.417 mg/mL MX combined with 3 mM or 5 mM H_2_O_2_, for 1 h, at 30 °C and 150 rpm. Cells grown in YPD containing only 1 mM or 5 mM H_2_O_2_ were used as control. Cell viability was assessed by CFU counting. Results were represented in graphs as the ratio of cells exposed to MX + H_2_O_2_ normalized to the cells exposed to H_2_O_2_. For the survival analysis, cells of the WT and mutant strains were incubated in liquid YPD medium for 1 h, at 30 °C, and 150 rpm in the absence (control) or in the presence of MX (0, 0.417, 0.834 or 1.67 mg/mL). Cells were then PBS-washed and the number of CFUs was assessed. For the analysis of virulence factors after MX exposal, cells from each strain were incubated with 0.417 mg/mL MX for 1 h, at 37 °C, PBS-washed, diluted and inoculated for melanin production and urease activity analyses. Visual detection was performed daily and documented after 72, 96 and 144 h of growth. The assay was performed as three independent experiments.

### 2.7. MIC Determination Assays

A stock solution of each antifungal agent was prepared as recommended by the NCCLS M27-A3 protocol. The following ranges were tested: Amphotericin B (Sigma-Aldrich, St. Louis, MO, USA) 0.125 to 16 μg/mL; Fluconazole (Sigma-Aldrich, St. Louis, MO, USA) 0.008 to 64 μg/mL. methoxy-amine was tested in the range of 0.125 to 16 mg/mL. One hundred μL of each chemical agent, diluted in RMPI medium, at a two-fold concentration, was placed in duplicate wells of sterile 96-well plates (Corning Glass Works, Corning, NY, USA).

An aliquot of 5 × 10^4^ yeast cells/mL in RPMI medium was added to the wells containing the drugs. Plates were then incubated at 37 °C, protected from light. End-points were macroscopically checked after 72 h. MIC (minimum inhibitory concentration) was defined as the lowest concentration at which there was a visually complete inhibition of growth compared to controls. The assay was performed as three independent experiments.

### 2.8. Evaluation of Drug Interactions

The interaction of amphotericin B (AmpB) with methoxy-amine hydrochloride (MX) was evaluated by the microdilution checkerboard method employing the H99, *apn1*∆, *apn2*∆ and *apn1*∆*apn2*∆ strains [45]. The drug concentrations varied from five-fold dilutions below to four-fold above the estimated MIC. Fifty μL of each drug, AmpB and MX, at a two-fold concentration in RPMI medium were added to 96-well plates.

All the strains were grown in YPD broth and incubated for 20 h at 30 °C with shaking. Cells were washed with 1× PBS and the inoculum concentration was adjusted to 1 × 10^5^ cells mL^−1^. One hundred μL of this solution was added to the wells containing 50 μL of both drugs at the 2× concentration. Positive controls without drugs and negative controls without yeast cells were performed. Plates were protected from light and incubated at 37 °C. After 72 h of incubation, endpoint cultures were visually read. Tests were performed in two biological replicates.

Cell proliferation was evaluated and fractional inhibitory concentration index (FICI) was calculated as previously described by Pfaller et al., (2015) [45]. FICI was calculated by the equation: FICI = (MICa in combination/MICa tested alone) + (MICb in combination/MICb tested alone); MICa: AmpB and MICb: MX. The result of the FICI was interpreted as follows: ≤0.5-synergy, >0.5 and ≤4.0-indifferent, >4.0-antagonism [45].

### 2.9. In Vitro Phagocytosis Assays

#### 2.9.1. Ethics Statement

All the animal procedures were performed in accordance with national and institutional guidelines for animal care and were approved by the University of Brasilia (UnB) Committee of Ethical Use of Animals (Proc. UnB Doc 52657/2011).

#### 2.9.2. Infection of Bone Marrow-Derived Macrophages

Bone marrow-derived macrophages (BMDMs) were obtained as previously described [46], by extracting the bone marrow from femurs and tibiae of C57BL/6 mice 8 to 12 weeks old (Animal Facility of the Institute of Biological Sciences of the University of Brasilia). Cells were cultured in vitro on non-treated 100 mm Petri dishes in complete RPMI 1640 medium (Thermo Fisher Scientific, Waltham, MA, USA) supplemented with 10% heat-inactivated fetal bovine serum (Thermo Fisher Scientific, Waltham, MA, USA), 50 µg/mL of gentamicin, 50 µM 2-mercaptoethanol (Sigma-Aldrich, St. Louis, MO, USA) and 20 ng/mL recombinant GM-CSF (PeproTech; Ribeirão Preto, SP, Brazil). The cultures were incubated for 8 days at 37 °C in a humidified 5% CO_2_ atmosphere. On day 3, 10 mL of fresh complete medium was added to the culture. Half of the medium volume was substituted on day 6. On day 8, BMDMs were detached from plates with TrypLE™ Express (Thermo Fisher Scientific, Waltham, MA, USA) and collected.

#### 2.9.3. Phagocytosis Assay

Approximately 5 × 10^4^ BMDMs were plated onto each well of 96-well polystyrene microplates with 100 μL of RPMI medium supplemented with 10% FBS. The cultures were incubated for 24 h at 37 °C in a humidified 5% CO_2_ atmosphere. The supernatant was removed and 2.5 × 10^5^ yeast cells of *C. neoformans* (Multiplicity of Infection; MOI = 5) were added to each well in 100 μL of RPMI medium supplemented with 10% FBS and the 18B7 mAb (kindly donated by Dr. Arturo Casadevall, Johns Hopkins University, Baltimore, MD, USA) at 10 μg/mL. The co-cultures of *C. neoformans* strains with macrophages were then incubated for 2 or 24 h post-infection (hpi) at 37 °C in a humidified 5% CO_2_ atmosphere. After 2 h of interaction, non-internalized yeast cells were removed by rinsing the wells with PBS. For the 24 h assays, fresh pre-warmed RPMI medium with 10% FBS was added to the wells.

For the quantitation of phagocytosis, cells were fixed and stained with Fast Panoptic kit (Laborclin, Pinhais, PR, Brazil). Under the Axio Observer Primovert GmbH microscope (Carl Zeiss Microscopy, Jena, Germany), 200 macrophages per well were observed for the determination of the percentage of phagocytosis (percentage of macrophages that have phagocytized one or more yeasts relative to the total number of cells counted) and the phagocytic index (the average number of phagocytized yeast cells per BMDM).

To determine the ability of the *C. neoformans* strains to proliferate inside BMDMs, colony-forming unit (CFU) assays were performed after 2 or 24 h of co-culture. After the incubation periods, yeast cells were released by lysing the macrophages with 0.05% SDS (Sodium dodecyl sulfate). For the 24 h assay, the supernatant and lysate were pooled. The number of CFUs was determined by serial dilution of each sample plated on YPD agar incubated at 30 °C and counted after 48 h. The phagocytic activity, defined as the number of CFUs obtained from a well at the two-hour time point, and intracellular growth, defined as the number of CFUs from the 24 h time point, were determined.

Three independent experiments were performed in technical triplicate. The multiple group comparisons were conducted by one-way analysis of variance (ANOVA) followed by Dunnett’s post-test. For the comparison of proportions, the Chi-square test was performed with a *p*-value <0.05.

### 2.10. Wax Moth Larvae Infection

*G. mellonella* infection was performed as previously described by Garciá-Rodas et al. [47]. Briefly, 16 randomly collected *G. mellonella* larvae were inoculated with 10 μL of a 10^6^ yeast cells/mL suspension by injection in the last left pro-leg using a sterile 26-gauge needle-fitted Hamilton syringe. *G. mellonella* larvae were inoculated with 1× PBS as control for the physical injury. Larval survival was daily evaluated for 7 days. Larval death was defined by the absence of movement in response to touch.

### 2.11. Statistical Analyses

Data are presented as the mean ± standard deviation (SD) unless otherwise stated. The statistical analysis was performed using software GraphPad Prism version 6.0 for Windows (GraphPad Software, San Diego, CA, USA, www.graphpad.com, accessed on 11 February 2021). The appropriate test is indicated for each experiment. *p* values ≤0.05 were considered statistically significant.

## 3. Results

### 3.1. Apn2-Like C. neoformans Is an ExoIII/Ape1 Member of the AP Endonucleases Family

**Figure 1:**
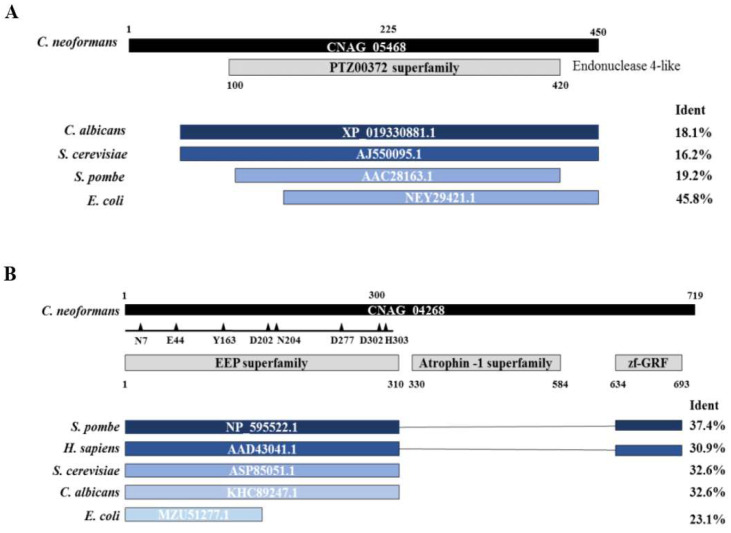
Comparative analysis of Apn deduced amino acid sequences. *APN1*-like (CNAG_05468) and *APN2*-like (CNAG_04268) gene sequences from *C. neoformans* were used as query for Blastp analyses. (**A**) Alignment results of Apn1 amino acid sequences from *C. albicans*, *S. pombe*, *S. cerevisiae* and *E. coli*, highlighting the cover and identity with the *C. neoformans* Apn1-like amino acid sequence. (**B**) Alignment results of Apn2 sequences from *S. pombe*, *H. sapiens*, *C. albicans*, *S. cerevisiae* and *E. coli*, highlighting the cover and identity with the *C. neoformans* Apn2-like deduced amino acid sequences. NCBI accession numbers are depicted in each protein representation. Triangles represent key-amino acids for the catalytic sites; Ident: percentage of amino acid identity provided by Blastp analysis on NCBI. EEP: exonuclease-endonuclease-phosphatase. zf-GRF: zinc finger domain.

The nucleotide sequences of *S. cerevisiae* ScApn1 (NCBI ID 853746) and ScApn2 (NCBI ID 852262) were used as queries for the NCBI BLASTp search against six-frame translations of the *C. neoformans* sequence database. The sequences CNAG_05468 and CNAG_04268 of the *C. neoformans* genome presented the highest identity with ScApn1 and ScApn2 sequences, respectively. *C. neoformans* Apn1 (CnApn1) predicted protein contains 483 amino acid residues, compared to 719 aa of the putative CnApn2 protein. Both deducted sequences share only 22.51% identity. The predicted CnApn1 protein presents two discrete domains: a xylose isomerase-like domain (position 138), and an nfo-apurinic endonuclease domain (endonuclease IV-like protein; position 428). The CnApn1 putative protein presents 48.3% identity with the Apn1 of *S. pombe*, 45.8% with the *E. coli* Endonuclease IV, 43.9% with the Apn1 of *C. albicans* and 42.4% identity with the *S. cerevisiae* Apn1 (Figure 1A).

The sequence CNAG_04268, encoding a putative exodes-oxyribonuclease III, was defined as the putative Apn2 of *C. neoformans*, and used as a query for BLASTp analyses on NCBI against the genomes of *S. pombe*, *Homo sapiens*, *S. cerevisiae, C*. *albicans,* and *E*. *coli* (Figure 1B). The predicted *C. neoformans* Apn2 shares several conserved and essential amino acid residues with *S. pombe* Apn2, human APE2 and *S. cerevisiae* Apn2.

The N-terminal region of the Apn2-like protein contains an Exonuclease–Endonuclease–Phosphatase (EEP) superfamily domain, conserved in all the analyzed species. This large superfamily includes the catalytic domain of a diverse set of proteins, including the ExoIII family of AP endonucleases [48]. In contrast to the Apn2 proteins from other organisms, the putative *C*. *neoformans* Apn2 presents an Atrophin-1 domain. Atrophin proteins are conserved transcriptional corepressors involved in nuclear receptor signaling [49]. About 250 amino acid residues, corresponding to the atrophin-1 superfamily domain of Apn2, are missing in the Apn1-like protein (Figure 1A).

### 3.2. apn1Δ, apn2Δ and apn1Δapn2Δ Mutant Strains Do Not Differ from the Parental Strain Regarding Osmotic or Oxidative Stress Sensitivity

AP-endonucleases knockout mutants were generated by homologous recombination after biolistic transformation of the H99 strain. To confirm the precise replacement of the *APN1* and *APN2* loci with the selective marker, multiple combinations of PCR primers were used (Figure S1A,B). The double mutant *apn1*Δ*apn2*Δ strain was also obtained. In order to verify if eventual phenotype alterations were indeed due to the deletion of the DNA sequences, *APN1* and *APN2* putative genes were reintroduced into the mutant strain’s genome, to create the reconstituted strains. The reintroduction of the sequences into the mutant strains genomes was confirmed by PCR (Figure S1A,B).

Initially, we evaluated if the putative *APN1* and *APN2* genes affected growth or viability of *C. neoformans* yeast cells. We observed no effect of the single or the double deletions on the growth in solid YPD or on their growth kinetics in liquid YPD broth at 30 or at 37 °C in comparison to the WT strain (Figures S2 and S3). To evaluate the potential role of *APN*-like genes for *C. neoformans* survival under stress conditions, *apn1*Δ, *apn2*Δ and the *apn1*Δ*apn2*Δ double mutant strains were tested for stress-related phenotypes. Overall, disruption of *APN1*, *APN2* or of both genes simultaneously induced mild defective phenotypes in response to cell surface stressors (sodium dodecyl sulfate, Sorbitol, caffeine), but none of the genotoxic (Cadmium sulfate (CaSO_4_) or osmotic (sodium chloride (NaCl) potassium chloride (KCl)) stressors, at the tested concentrations, affected the growth of the *C. neoformans* mutant strains at 30 or 37 °C (Figure S2A,B). Additionally, we did not observe any significant difference in the mutant’s response to oxidative stress induced by menadione (MND), sodium nitrite (NaNO_2_) or hydrogen peroxide (H_2_O_2_) (Figure S4). These data demonstrate that the putative *APN1* and *APN2* sequences are not essential for growth or response of *C. neoformans* to those stressors.

### 3.3. C. neoformans APN1 and APN2-Like Genes Are Involved in the Repair of DNA Radiation-Induced Lesions

**Figure 2:**
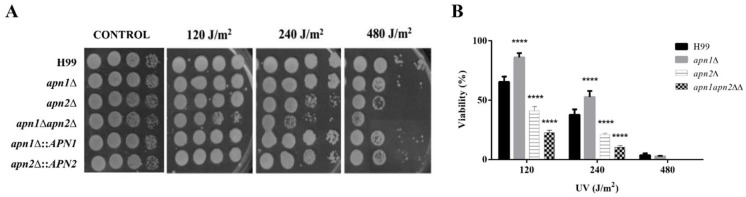
Disruption of Apn1- and Apn2-like genes induced *C. neoformans* susceptibility to UV radiation. (**A**) Ten-fold serial dilutions of *C. neoformans* WT, mutant and reconstituted strains of yeasts were exposed to 120, 240 and 480 J/m^2^ doses of UV radiation (254 nm) and grown at 30 °C, for 48 h. The results are representative of at least three independent experiments. (**B**) Cell viability (%) after UV irradiation. *C. neoformans* 5 × 10^2^ cells were plated on YPD agar and exposed to UV radiation at 120, 240 and 480 J/m^2^ at 254 nm. The plates were incubated at 30 °C for 48 h, protected from light, for colony forming unit (CFU) counting. Graph represents the percentage of viable cells normalized by the percentage of viable control cells. The WT H99 strain was used as control for statistical comparison. One-way analysis of variance (ANOVA) with Dunnett’s post-test was used to compare the means of results from three independent experiments. Error bars represent standard errors of the mean. **** *p*  <  0.0001.

To assess if the CnApn1 and CnApn2 predicted proteins participate in the repair of radiation-derived DNA lesions, WT and mutant strains were exposed to different UV radiation dosages, and the viability of fungal cells was evaluated. Disruption of *APN1* and *APN2*-like sequences resulted in increased susceptibility of *C. neoformans* to UV radiation (Figure 2). Deletion of both the *APN*-like genes induced a higher sensitivity to UV radiation when compared to the single mutant strains’ phenotypes (Figure 2A). The susceptibility of the strains to UV radiation was also investigated by plating on YPD agar a defined number of cells after irradiation with 120, 240 and 480 J/m^2^ at 254 nm. Yeasts’ viability was estimated by CFU counting, and the results indicated that the disruption of *APN2* significantly affected the *C. neoformans* survival in a radiation dose-dependent manner (Figure 2B). Even though moderate resistance to UV radiation was observed for the *apn1*∆ strain when compared to the WT, the double deletion of *APN1* and *APN2* promoted an even higher sensitivity to UV radiation than the *APN2* single mutation (Figure 2B).

### 3.4. The Putative Apn2 Endonuclease Is Involved in Processing Alkylation-Induced DNA Damage in C. neoformans

**Figure 3:**
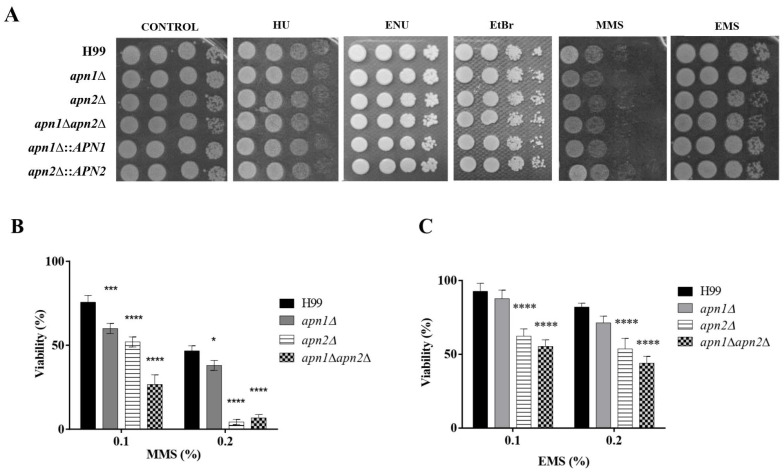
Predicted Apn2 endonuclease is required for yeast viability after exposure to DNA-alkylating agents. (**A**) The different strains were serially diluted ten-fold and plated onto YPD agar supplemented with DNA damage-inducing agents: 0.03% methyl methane-sulfonate (MMS), 0.04% ethyl methane-sulfonate (EMS), 200 µg/mL N-ethyl-N-nitrosourea (ENU), 20 µg/mL ethidium bromide (EtBr) or 110 mM hydroxyurea (HU). The plates were incubated at 30 °C for 48 h. The results are representative of three independent experiments. (**B**) Cell viability after exposure to 0.1 or 0.2% MMS or (**C**) 0.1 or 0.2% EMS. The strains were exposed to the agents in liquid YPD for 1 h, at 30 °C and 150 rpm. Cells were then phosphate-buffer saline- (PBS)-washed and plated on YPD agar for CFU counting. The graphs represent the percentage of viable cells normalized by the percentage of viable control cells. The WT H99 strain was used as control for statistical comparison. One-way ANOVA with Dunnett’s multiple-comparisons test was used to compare the means of results from three independent experiments. Error bars represent standard errors of the mean. **** *p*  <  0.0001; *** *p*  <  0.001; * *p*  <  0.05.

In order to evaluate the possible roles of CnApn1 and CnApn2 predicted proteins in the repair of different types of DNA lesions, the growth of the mutant strains was evaluated after their exposure to different mutagenic agents. Neither the single mutant (*apn1*Δ and *apn2*Δ) nor the double mutant (*apn1*∆*apn2*∆) strains displayed any growth difference in comparison to the WT strain after exposure to hydroxyurea (HU, 110mM), the alkylating compounds methyl methane-sulfonate (MMS, 0.03%), ethyl methane-sulfonate (EMS, 0.04%) and N-ethyl-N-nitrosourea (ENU, 200 µg/mL), or the intercalating agent ethidium bromide (EtBr, 20 µg/mL) (Figure 3A).

We further submitted the mutants to higher concentrations of MMS and/or EMS (0.1% or 0.2%) for 1h, at 30 °C, followed by CFU counting to access yeasts’ viability (Figure 3B). Cells from the *apn1*∆ single mutant were only sensitive to MMS-induced stress at both tested concentrations. At 0.2% MMS, the survival of *C. neoformans apn2*∆ was drastically reduced to less than 10% in comparison to the wild-type cells (75% survival). Treatment with EMS also promoted, to a lesser extent than MMS, a reduced viability of the *apn2*∆ mutant in relation to the WT strain. In comparison to the wild-type and single-gene deletion strains, the *apn1*∆*apn2*∆ double mutant was more sensitive to higher concentrations of MMS and EMS. These results indicate a major involvement of CnApn2 in the response to MMS and EMS-induced alkylated DNA.

### 3.5. The Deletion of APN-Like Genes Results in Increased Sensitivity to Zeocin and DNA Fragmentation

**Figure 4:**
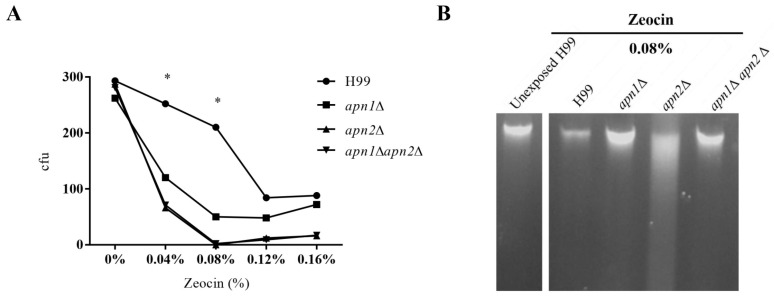
The ablation of the predicted Apn1 and Apn2 proteins sensitized *C. neoformans* yeasts to the exposure to Zeocin. (**A**) Cell viability of the WT and mutant strains after incubation with different concentrations of Zeocin. Yeasts from WT and mutant strains were cultured in liquid YPD containing 0.04%, 0.08%, 0.12% or 0.16% Zeocin for 1 h, at 30 °C, 150 rpm. Cells were then PBS-washed and plated on YPD agar for CFU counting. Percent of viable cells was calculated from the CFU counting. Data are presented as mean of three replicates. Statistical test: One-way ANOVA with Dunnett’s post-test, * *p* < 0.05. (**B**) Electrophoretic analysis in 1% agarose gel stained with 0.5 µg/mL EtBr of *C*. *neoformans* strains DNA after exposure to 0.08% Zeocin for 1 h. Unexposed mutant and H99 strains presented DNA with no fragmentation. Genomic DNA from the unexposed H99 strain was used as control. The result is representative of three independent experiments.

Zeocin is a genotoxic drug that intercalates into the DNA, provoking double-strand breaks [50]. To evaluate the influence of the predicted Apn proteins in the response of *C. neoformans* to Zeocin, the mutant and WT strains were exposed to different concentrations of the drug, for 1h. The deletion of *APN1* and *APN2* resulted in increased sensitivity to Zeocin even in low doses (0.04% or 0.08%) (Figure 4A).

To investigate the possible contribution of the putative AP-endonucleases in the protection of *C. neoformans* DNA from the damage provoked by Zeocin, we qualitatively analyzed the DNA fragmentation in the WT, *apn1∆*, *apn2∆* and *apn*1*∆apn2∆* mutant strains. Unexposed and (0 h) control cells showed almost undetectable levels of DNA fragmentation (Figure 4B). Nonetheless, DNA smearing was observed after 1 h of exposure to 0.08% Zeocin for all the strains (Figure 4B). The *apn2∆* mutant displayed a marked increase in Zeocin-induced DNA fragmentation in comparison to the WT (Figure 4B). The increase in DNA fragmentation in *apn2∆* suggests a reduced DNA repair capability.

### 3.6. The Inhibition of Recognition of Abasic Sites by Methoxy-Amine Enhances the Fungicidal Effect of Amphotericin B

Amphotericin B (AmpB) generation of reactive oxygen species contributes to the fungicidal effect of this drug [49]. We then decided to evaluate whether compounds that inhibit the corrections of oxidative lesions, such as the anti-cancer chemotherapeutic drug methoxy-amine (MX), could increase the antifungal effect of AmpB. We first determined the MIC values for MX, AmpB and Fluconazole alone for each strain (Table 1). The mutant strains *apn2*Δ and *apn1*Δ*apn2*Δ presented MIC values of 1.0 µg/mL of Fluconazole, half of the MIC values presented by the H99, *apn1*Δ and reconstituted strains (2.0 µg/mL). Based on these values, we used different combinations of the MX and AmpB drugs for a checkerboard assay, to evaluate a possible drug interaction. The combination of AmpB and MX showed synergism against all the strains. The combination of these drugs was more effective against the *apn2*∆ (FICI = 0.187) and *apn1*∆*apn2*∆ (FICI = 0.219) strains than for H99 (FICI = 0.375) and *apn1*∆ (FICI = 0.312) (Table 2).

**Figure 5:**
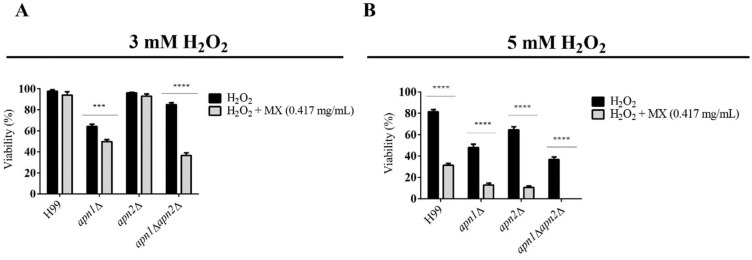
Methoxy-amine (MX) enhances the effects of H_2_O_2_ on the viability of the *C*. *neoformans apn*1Δ*apn*2Δ mutant strain yeasts. Cell survival of *C*. *neoformans* WT and mutant strains for the combined exposure to MX and H_2_O_2_ Strains were cultured in liquid YPD containing 0.417 mg/mL MX and (**A**) 3 or (**B**) 5 mM H_2_O_2_, for 1 h, at 30 °C and 150 rpm. Cells incubated only in YPD containing 3 or 5 mM H_2_O_2_ were used as comparison. The graph represents the percent of survival of each strain exposed to each condition described compared to the control condition (YPD medium). Two-way ANOVA with Sidak’s multiple-comparisons test was used to compare the means of results from three independent experiments. Error bars represent standard errors of the mean. **** *p*  <  0.0001; *** *p*  <  0.001.

Since MX inhibits the recognition and correction of abasic sites by AP-endonucleases, we investigated the effect of MX exposure in combination with H_2_O_2_-induced oxidative stress in WT and *APN*-like genes’ mutant strains (Figure 5). The strains were exposed to 3 mM or 5 mM H_2_O_2_ in combination with 0.417 mg/mL MX, for 1 h, at 30 °C and 150 rpm. Yeasts’ viability was assessed by CFU counting. The exposure to 3 mM H_2_O_2_ combined with MX provoked a significant decrease in cell viability for the *apn1*Δ and the *apn1*Δ*apn2*Δ mutant strains (Figure 5B), but not *apn2*Δ. After exposure to 5mM H_2_O_2_ and MX, there was a marked reduction of CFU recovery for all the strains.

### 3.7. The CnApn1 and CnApn2-Like Sequences Are Required for the Production of Melanin

**Figure 6:**
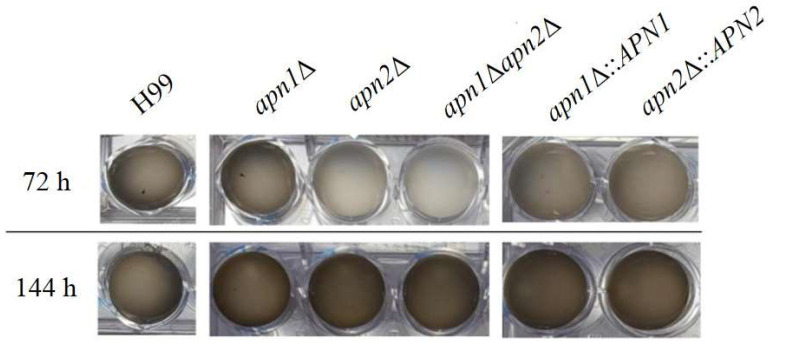
The disruption of the Apn2-like gene of *C. neoformans* impairs melanin production. *C. neoformans* yeasts were incubated in melanin-inducing minimal medium with 1 mM l-DOPA at 37 °C, 150 rpm, protected from light. Melanin production was visually assessed by the medium color change from translucent to brown and photo-documented at 72 and 144 h. The result is representative of three independent experiments.

We also evaluated the involvement of the predicted BER proteins CnApn1 and CnApn2 in the expression of some virulence-associated phenotypes of *C. neoformans* in vitro, such as the polysaccharide capsule, phospholipase and urease activities and melanization. We did not observe any defect in the capsule expansion after growth on inducing conditions at 30 or 37 °C (Figure S5A), phospholipase (Figure S5B) or urease activity (Figure S5C) in the mutant strains. However, deletion of *APN2,* but not of *APN1*, induced a marked delay in melanin production by *C. neoformans* at 37 °C in 1 mM l-DOPA-inducing medium (Figure 6). Prolonged incubation (144 h) resulted in late melanization of the *apn2*∆ and *apn1*∆*apn2*∆ strains. This defect did not seem to be related to impaired cell growth or density, because there were no significant differences in CFU counts at 72 or 144 h after melanin synthesis among the strains (data not shown). The delayed melanin synthesis was reversed in the *apn2*∆::*APN2* reconstituted strain (Figure 6).

### 3.8. The Recognition and Correction of Abasic Sites in DNA Is Required for Proper Melanin Production by C. Neoformans

**Figure 7:**
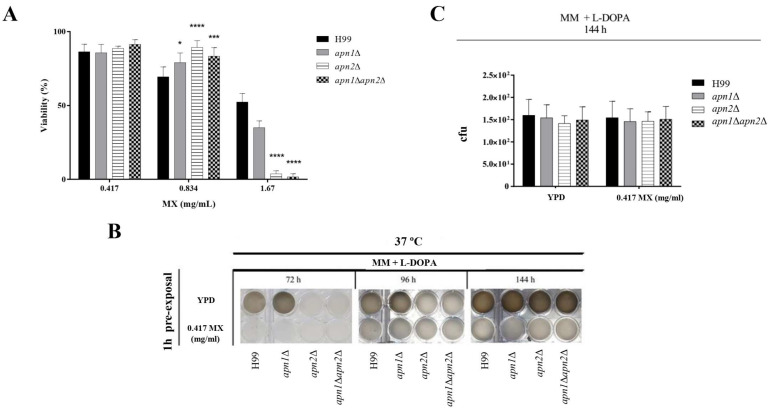
Apn1- and Apn2-like genes are required for melanin production by *C. neoformans*. (**A**) Viability of *C. neoformans* cells after exposure to methoxy-amine (MX). Cells of the WT and mutant strains were incubated in liquid YPD medium for 1 h, at 30 °C, and 150 rpm in the absence (control) or in the presence of MX (0, 0.417, 0.83 or 1.67 mg/mL). Cells were then PBS-washed and the number of CFUs was assessed. The graph represents the percent of survival of each strain exposed to MX compared to the control condition. The wild-type H99 was used as a control group for statistical comparison. One-way ANOVA with Dunnett post-test was used to compare the means of results from three independent experiments. Error bars represent standard errors of the mean. **** *p*  <  0.0001; *** *p*  <  0.001; * *p*  <  0.05. (**B**) Melanin production after exposure of yeast cells to MX. Cells from each strain were incubated with 0.417 mg/mL MX for 1 h, at 37 °C, PBS-washed, diluted and inoculated for melanin production analyses. Visual detection was performed daily and photo-documented after 72, 96 and 144 h of growth. Results are representative of three independent experiments. (**C**) Cell viability of *C. neoformans* strains after MX exposure and induction of melanin synthesis. Aliquots of cell suspensions were collected from the melanin assay plates after 144 h of incubation, and viable colonies were assessed by CFU counting. One-way ANOVA with Dunnett’s post-test was used to compare the means of results from three independent experiments.

To confirm the role of *C. neoformans* Apn2 in melanin production, we decided to investigate the effects of MX presence during the induction of melanin synthesis. First, we evaluated possible effects on MX on fungal viability by treating cells of the different strains with 0.417, 0.834 or 1.67 mM of this compound for 1 h at 30 °C. As, at 0.417 mg/mL, this drug was not lethal to either the WT or mutant strains (Figure 7A), this concentration was chosen for the next experiments. The different strains were exposed to 0.417 mg/mL MX, washed and subject to melanin-inducing conditions. The pre-treatment of *C. neoformans* with MX delayed and reduced the melanization levels of the wild-type strain (Figure 7B), resulting in a phenotype similar to the one observed for the unexposed *apn2*∆ and *apn1*∆*apn2*∆ mutant strains under inducing conditions at 37 °C (Figure 7B). These effects were not associated with cell death, because there was no differences in CFU counts between untreated and treated samples (Figure 7C). Even after 10 days of incubation, the melanization levels of MX-treated wild-type or mutant strains did not increase (data not shown).

### 3.9. Predicted C. neoformans Apn1 and Apn2 Proteins Influence Phagocytosis and Fungal Survival within Macrophages In Vitro

**Figure 8:**
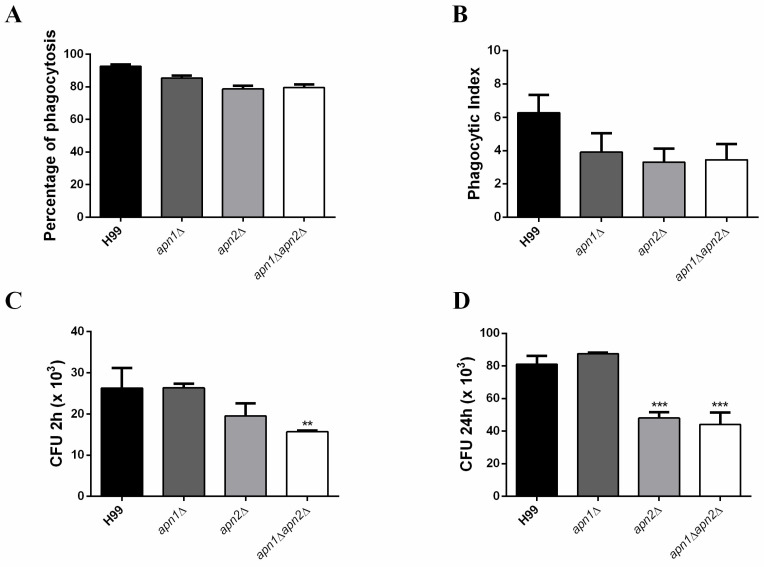
The deletion of APN-like genes results in *C. neoformans* reduced phagocytosis and survival in macrophages in vitro. Bone marrow-derived macrophages (BMDMs) were co-cultured for 2 h or 24 h with previously opsonized fungal cells from the different strains of *C. neoformans*. Each strain was inoculated into three wells of a 96-well polystyrene plate containing macrophages at a MOI of 5. (**A**) Percentage of phagocytosis. Data are presented as mean ±95% C.I. (*n* = 3 experiments, *** *p* < 0.001). (**B**) Phagocytic index. (**C**) CFU counts of *C. neoformans* strains recovered from bone marrow-derived macrophages (BMDMs) after 2 (**D**) or 24 h of co-incubation. Error bars represent standard errors of the mean of three biological replicate experiments. The wild-type H99 was used as a control group for statistical comparison. Statistical test: One-way ANOVA with Dunnett’s post-test, ** *p* < 0.01.

Phagocytes play multiple roles in cryptococcal pathogenesis, and the ability to survive and proliferate inside phagocytes is associated with patient outcome [51]. To access the impact of Apn1 and Apn2 on *C. neoformans* virulence, we evaluated the in vitro interaction of the mutant strains with macrophages (activated BMDMs) by means of phagocytosis and killing assays. We observed a decrease in the rates of internalization and in the total number of internalized yeast cells from mutant strains in comparison to the WT (Figure 8A,B). In addition, the deletion of the *APN2*-like gene led to a reduced number of viable *C. neoformans* cells recovered from the macrophages (Figure 8C,D). Similar results were obtained for the *apn1*∆*apn2*∆ double mutant. In contrast, the absence of the *APN1*-like gene did not change the number of fungal CFUs after the interaction.

### 3.10. Deletion of APN Genes Provokes Minor Effects in the Wax Moth Larva Model of Infection

In vivo effects of the deletion of *APN*-like genes in *C. neoformans* virulence were accessed by infection of *Galleria mellonella* larvae. Infection with both *apn2*∆ and *apn1*∆*apn2*∆ strain resulted in slightly delayed killing kinetics compared to the wild type (Figure S6). The disruption of *APN1* did not alter fungal virulence in this model.

## 4. Discussion

In this work, we examined the role of two BER pathway Apn-like sequences in the proliferation, stress response and virulence of the human pathogen *C. neoformans*. We identified two genes encoding putative Apurinic/apyrimidinic (AP) endonucleases in this fungus’ genome. The CnApn1 (CNAG_05468) predicted protein possesses the nfo domain (Exonuclease IV family), and is consistently different from CnApn2, an Xth family protein (Figure 1B). From the sequences analyzed in this study, only the Apn2 proteins from *C. neoformans*, *S. pombe* and *H. sapiens* possess a zf-GRF domain. The presence of the Athrofin-1 and zf-GRF domains indicates a transcription regulation activity for the deducted proteins [48,49]. The absence of this domain in the predicted CnApn1 and in the Apn2 proteins of other fungi suggests that Apn proteins might display distinct roles in DNA repair and cellular responses in yeasts.

*C. neoformans* (here CnApn2) was previously suggested to be a transcription factor after a screening performed by Jung et al. (2015) [52]. The authors evaluated the presence of DNA sequences associated with transcriptional activity in the *C. neoformans* genome database, detected that the CNAG_04268 null mutant had a growth defect at 39 °C and further characterized the potential role of this gene as transcription factor [52]. However, the authors did not discuss the homology of the CNAG_04268 deducted protein with DNA repair components, nor did they evaluate the effect of combined disruption of Apn1 and Apn2 in the context of DNA damage-response and survival of *C. neoformans*.

To investigate the potential roles of the predicted AP-endonucleases in the biology of *C. neoformans*, we constructed null mutant strains in which the *APN1* and *APN2* putative genes were deleted. The deletion of *APN*-like genes individually or simultaneously indicated that none of the *APN* putative genes is essential for *C. neoformans* growth under standard laboratory conditions. The disruption of those genes did not affect the response of *C. neoformans* to cell wall or membrane stressors (Figure S2). Similar results were previously reported in *S. cerevisiae*, *C. albicans* and *S. pombe* [19,22,23].

In addition, deletion of Cn*APN1* and Cn*APN2* putative genes did not affect the sensitivity of *C. neoformans* to different sources of oxidative stress (H_2_O_2_, MND or NaNO_2_) (Figure 2) as previously observed for *C. albicans apn1*Δ mutants exposed to H_2_O_2_ [22]. In contrast, the *apn2*Δ mutant was slightly sensitive to H_2_O_2_ [23,53]. In *S. cerevisiae*, the simultaneous ablation of the Ntg1, Ntg2, and Apn1 BER proteins resulted in increased recombination and mutation rates but did not influence the response to the oxidizing agents H_2_O_2_ and Menadione [54].

We demonstrated that *C. neoformans APN* deletions induced increased sensitivity to MMS, EMS, MX and UV radiation in a dose-dependent way (Figure 3 and Figure 4). Notably, those agents are known to induce SSB (Single Strand Breaks) and DSB (Double Strand Breaks) on DNA. In contrast, a previous study reported that *C. albicans* BER and NER mutants exhibited a WT sensitivity to EMS and MMS [22,55], suggesting that the BER and NER pathways do not play a major role in repairing DNA breaks in this organism [22,55]. They also observed that *APN2* deletion in *S. cerevisiae* did not affect its response to MMS-induced damage, but the *apn1*∆*apn2*∆ double mutant displayed a marked increased sensitivity to MMS in comparison to the *apn1*∆ strain [22,55].

In the *S. pombe* BER pathway, mutant strains *nth*1Δ and *apn2*Δ were sensitive to MMS while the *apn1*Δ strain was also sensitive to oxidative stress. *S. pombe* *apn2*Δ*apn1*Δ double mutant was more sensitive to the oxidative stress than the *apn2*Δ single mutant [23]. Disruption of *APN2* in *S. pombe* also induced sensitivity to phleomycin, which indicates Apn2 as the major AP-endonuclease of this fungus [23].

We also observed that deletion of the *C. neoformans APN2*-like gene resulted in altered responses to alkylating agents, when compared to the WT and *apn1*∆ strains. The *C. neoformans apn2*Δ strain also presented reduced viability and increased genomic DNA fragmentation after exposure to Zeocin, a Bleomycin family drug (Figure 5). The *apn1*Δ mutant still possesses the *APN2* gene and presented the WT-type phenotype in response to Zeocin exposure. Taken together, these data suggest that Apn2 is the major contributor not only to the AP-endonuclease but also to the 3′-phosphodiesterase activity in *C. neoformans*. Curiously, although the Zeocin exposure induced significant death in all mutant strains, it did not provoke DNA fragmentation for the *apn1*∆*apn2*∆ double mutant strains. This suggests that the mechanism of death induced by Zeocin may be different for this mutant, not necessarily inducing major DNA fragmentation, such as by accumulation of abasic sites, by blocking RNA transcription and/or by cell cycle arrest.

The differences observed in the BER mutant strains’ responses to UV radiation and alkylating agents of *C. albicans* and *S. cerevisiae* compared to *C. neoformans* and *S. pombe* could be associated with the proteins’ structure. The human homologues of the yeast Apn proteins are involved in the repair of DSB of DNA [56,57] and HsAPE2 presents a robust 3′→5′ exonuclease activity towards mismatched 3′-terminal bases, as well as 3′-phosphoglicolate removal activity [57,58,59]. The *S. pombe* Apn2 and the *C. neoformans* predicted Apn2 protein both present the same zf-GRF domain described as important for the HsAPE2 3′ exonuclease activity, which is absent in the related proteins of *C. albicans* and *S. cerevisiae*, and also in CnApn1 (Figure 1). Our observations suggest that the predicted AP-endonuclease 2 of *C. neoformans* potentially plays a role in additional DNA repair pathways, beyond BER, probably acting in the recognition and correction of SSB and DSB on DNA.

The differences between CnApn1 and CnApn2 sequence domains are probably the reason for the consistent distinct phenotypes of Apn single mutants in response to stress conditions. Apn1 possesses only an AP-endonuclease domain and *apn1*∆ mutants presented, in general, phenotypes similar to the WT strain. Only the dose-dependent response to lower doses of UV radiation and H_2_O_2_ + MX co-exposure phenotypes were specific for *apn1*∆.

The resistance to lower doses of UV radiation observed in the *apn1*∆ mutant strain could be associated with the reduction of DNA cleavage performed by Apn1. The absence of Apn1 could reduce the cleavage of DNA strands at AP-sites (a UV-radiation induced lesion) and consequently the formation of phosphodiester gaps in DNA strain. This mechanism can be associated with the data observed in Figure 5, when *apn2*∆ mutants presented a WT phenotype in response to 3 mM H_2_O_2_ + MX, a combination that induces base oxidation and AP-sites in DNA. The *apn2*∆ mutant strain still possesses active Apn1 proteins, whose activity could be sufficient to maintain the WT-phenotype in the *apn2*∆ mutant strain.

On the other hand, Apn2 possesses domains involved in biological processes that go beyond DNA repair. The *apn2*∆ strain was more susceptible to UV radiation, alkylation, oxidative and antibiotic stress. Apn2 was also listed as a transcription factor, so it is possible that some of the distinct phenotypes presented by the mutant strain result from the absence of Apn2 transcriptional activity. The *apn1*∆*apn2*∆ double mutant presented similar phenotypes to *apn2*∆, except for the DNA fragmentation pattern and the susceptibility to H_2_O_2_ with MX. The phenotypes observed for double mutants do not always correspond to the absence of individual genes, nor even to a synergic effect of both deletions. Therefore, it is important to note that the *apn1*∆*apn2*∆ mutant may have a specific mechanism of cellular response to stress in order to compensate for the disruption of the cellular AP- endonuclease activity.

Cryptococcosis affects thousands of people yearly and, although it is important to global health, therapeutic options are extremely limited [60]. We demonstrated that the deletion of the *APN2*-like gene slightly increased *C. neoformans’* susceptibility to fluconazole exposure, and that the simultaneous deletion of both predicted APN sequences increased the fungus’ sensitivity to AmpB and fluconazole in vitro. We also demonstrated that exposure of *C. neoformans* to the synthetic AP-site inhibitor MX in combination with AmpB significantly enhanced its antifungal effect, even for the WT strain (Table 2). MX also sensitized cells to the oxidative stress induced by H_2_O_2_ (Figure 5B).

DNA repair-inhibitor drugs, such as MX, are well established in clinical practice as adjuvants [32]. Given the emergence of antifungal drug-resistant strains and the increasing incidence of systemic and invasive mycoses [33,61,62], the identification of novel antifungal drug targets and adjuvant options is an urgent clinical need. We demonstrated the synergism between MX and AmpB, with an increased fungicidal effect on concentrations higher than 75 µg/mL of MX. Although 75 and 150 µg/mL of MX are higher concentrations than those used in clinical trials in cancer therapies (100 mg/m^2^ or 50 ng/mL of plasma) [63], our results are still promising. The synergic effect of MX and AmpB is an interesting starting point for further investigations and highlights the possibility of using DNA repair inhibitors as a class of adjuvants in antifungal therapy research.

The deposition of melanin in the cell wall is critical to the virulence of *C. neoformans* and other pathogenic fungi [64,65,66]. We demonstrated that the disruption of *APN*-like genes resulted in a delay and significantly lower levels of melanin production by *C. neoformans* (Figure 6) without compromising cell viability or growth rate. Melanin production is activated by oxidative stress conditions in several microorganisms [65,66]. In the absence of Apn activity, overlapping enzyme repair systems may be activated later to eliminate the DNA damage accumulation and proceed with melanin synthesis. This might explain the restored melanin production after 144 h hours in the mutant strains (Figure 6). According to our data, not only the AP-endonuclease activity of Apn proteins, but also the generation/repair of the abasic site itself, might play a role in melanin production by *C. neoformans*, since the pre-treatment of *C. neoformans* strains with MX for 1 h had a significant impact on the production of the pigment, even in the WT strain (Figure 7). MX is highly specific and rapidly binds to abasic sites on DNA. The MX–AP–site ligation is strong and stable [67]. Therefore, the phenotype of melanin production blockage is prolonged (10 days), and it is observed even in response to a short time of drug exposure.

*C. neoformans* is resistant to the host cell’s oxidative stress response, mainly due to the production of melanin and antioxidant compounds [37,39]. Several studies have demonstrated a correlation between the capacity of *C. neoformans* clinical strains to be phagocytosed, and/or to proliferate intracellularly, and poor patient outcomes [68,69,70]. Due to their potential role in melanization, we extended our analysis to evaluate the *APN*-like mutant’s virulence during their interaction with macrophages and *G. mellonella* larvae. Interestingly, both *C. neoformans APN*-like mutants were less efficiently internalized and more susceptible to killing by M2 macrophages in vitro (Figure 8). The involvement of DNA repair proteins in complex responses, such as interaction with phagocytes, was not exactly expected.

Santiago et al. (2015) used a mutant library screen to list genes involved in macrophage uptake and demonstrated several non-expected genes involved in pathogen-host interaction [51]. One of the listed genes was *APN1*, responsible for a decrease in macrophage up-take. However, the mechanism of how individual gene products modulate interactions with host phagocytes is still poorly understood. Since no significant difference was observed in capsule enlargement between *apn* mutants, other characteristics may be altered in these mutants to influence fungal internalization. We hypothesized the participation of Apn proteins in other cellular mechanisms, beyond DNA repair, as occurs for the mammalian homologue APE1 [17,71]. The impact of Apn proteins’ absence on fungal survival inside macrophages may be associated, even in part, with the delay in melanin production, sensitizing the cells to the oxidative stress induced by the phagocyte. This idea can also be the reason for the slight delay in *G. mellonella* killing by *apn2*∆ and *apn1*∆*apn2*∆ mutant strains (Figure S6), but much is still to be clarified.

## 5. Conclusions

This work demonstrated the impact of AP-endonucleases-like genes in the *C. neoformans* response to cell stress resistance and virulence. *C. neoformans* AP endonucleases-like genes were shown to be important in protecting the yeast cells against alkylation and UV-induced damage in vitro. The disruption of *C. neoformans* putative Apn proteins delayed the production of melanin, one of the most important virulence factors in this fungus. The melanin production defect seems to be related to the absence of AP-repair activity, since the exposure to MX completely abolished the pigment production. The MX sensitized *C. neoformans* to H_2_O_2_ exposure and boosted the fungicidal effect of AmpB, in a synergic way. The Apn-like activities were also required for the interaction and growth inside murine macrophages. This work highlighted the importance of DNA repair pathways for *C. neoformans’* response to cell stress conditions and the potential use of DNA repair inhibitors to improve the antimicrobial effect of antifungal drugs.

## Figures and Tables

**Table 1:** MIC values of amphotericin B (AmpB), methoxy-amine hydrochloride (MX) and Fluconazole (Flu) against H99, *apn1*∆, *apn2*∆ and *apn1*∆*apn2*∆ and reconstituted strains.

Strains	Replicate 1			Replicate 2		
	AmpB (µg/mL)	MX (mg/mL)	Flu (µg/mL)	AmpB (µg/mL)	MX (mg/mL)	Flu (µg/mL)
H99	0.25	1.25	2	0.25	1.25	2
*apn1*∆	0.25	1.25	2	0.25	1.25	2
*apn2*∆	0.125	0.626	1	0.125	0.626	1
*apn1*∆*apn2*∆	0.125	0.626	1	0.125	0.626	1
*apn1*∆::*APN1*	0.25	1.25	2	0.25	1.25	2
*apn2*∆::*APN2*	0.25	1.25	2	0.25	1.25	2

**Table 2:** MIC values of amphotericin B (AmpB) and methoxy-amine hydrochloride (MX) in combinations and fractional inhibitory concentration index (FICI) values.

Strains	Replicate 1			Replicate 2		
	MICab	MICba	FICI	MICab	MICba	FICI
H99	0.0625	0.292	0.50	0.0312	0.150	0.2498
*apn1*∆	0.0156	0.292	0.3124	0.0625	0.078	0.3125
*apn2*∆	0.0078	0.078	0.1874	0.0078	0.078	0.1874
*apn1*∆*apn2*∆	0.0156	0.078	0.2498	0.0078	0.078	0.1874

MICab: MIC of AmpB in combination with MX. MICba: MIC of MX in combination with AmpB. The AmpB concentrations are expressed in µg/mL, and for MX in mg/mL.

## Data Availability

The data that support the findings of this study are available from the corresponding authors upon reasonable request.

## Supplementary Materials

The following are available online at https://www.mdpi.com/2309-608X/7/2/133/s1, Figure S1: Confirmation of knockout mutants by genomic DNA PCR amplification, Figure S2: Temperature and chemical cell stress-related phenotypes of the *C*. *neoformans APN*-like genes’ mutants, Figure S3: Growth curve of *C. neoformans* WT and mutant strains, Figure S4: The *apn1∆*, *apn2∆* and *apn1∆apn2∆* mutants are not susceptible to oxidizing agents, Figure S5: The capsule expansion, phospholipase and urease activities were unaffected by the disruption of Apn-like genes, Figure S6: Evaluation of virulence of *C. neoformans APN* mutants using *G. mellonella* infection model, Table S1: Primers used in this study and Table S2: Strains used in this study.
