# Supplementary material for: Base Excision Repair AP-Endonucleases-Like Genes Modulate DNA Damage Response and Virulence of the Human Pathogen Cryptococcus neoformans
Source: J Fungi (Basel). 2021 Feb 12;7(2):133. doi: 10.3390/jof7020133 (PMC7917787; doi:10.3390/jof7020133)
Supplement: Supplementary file 1 [file jof-07-00133-s001.pdf]

Supplements – Oliveira, R.K.M. Base Excision Repair AP-Endonucleases-Like Genes Modulate DNA Damage Response and Virulence of the Human Pathogen *Cryptococcus neoformans*

Table S1. Primers used in this study

**Number**	**Identification**	**Sequence (5′→3′)**
APN1		
**P1(*APN1*)**	5'ext_APN1F	CGAGGAGCATAAGGAAGGAGA
**P10(*APN1*)**	3'ext_APN1R	GCATGACAACTTTCCCCTAGC
**P3 (*APN1*)**	5'APN1F	GAGTTAAGGGGTGGGAGGAAT
**P4 (*APN1*)**	3'APN1R	TCGCTGGAGTCTGTAGCTTGA
**P5 (*APN1*)**	nat_F	AGCAAGACCCATCAAAGCTCTA
**P6 (*APN1*)**	nat_R	CTCCAGAACATTCGTCGCTTAC
**P11(*APN1*)**	M13_5'APN1R	ATCATGTCATAGCTGTTTCCTGTCTGGGCCTTTCACTCAAGA C
**P12(*APN1*)**	M13F_3'APN1F	GCACTGGCCGTCGTTTTACCAGACACGGCAGCTATCATTC
APN2		
**P1(*APN2*)**	5'ext_APN2F	CAAGCACCTTCTTACCCTCCT
**P10(*APN2*)**	3'ext_APN2R	CCACTTACCAAAGCTCACTGG
**P3 (*APN2*)**	5'APN2_F	GAATCGGACGATGATGCTGT
**P4 (*APN2*)**	3'APN2R	CAAACCCCATTCCCTACTGAC
**P5 (*APN2*)**	hyg_F	GCGGGAGATGCAATAGGTCAG
**P6 (*APN2*)**	hyg_R	AGCTCTCGGAGGGCGAAGAAT
**P11(*APN2*)**	M13_5'APN2R	ATCATGTCATAGCTGTTTCCTGCTGTGTAGTGTCCCGATCCA T
**P12(*APN2*)**	M13F_3'APN2F	GCACTGGCCGTCGTTTTACCCCTTCTGGTTTAGCTGACG

Table S2. Strains used in this study

**Strain**	**Genotype**	**Source**
**H99**	*MAT α*	Perfect *et al.* (1980) [1]
apn1Δ	*apn1::nat MAT α*	This study
apn2Δ	*apn2::hyg MAT α*	This study
apn1Δapn2Δ	*apn1::nat apn2::hyg MAT α*	This study
apn1Δ::APN1	*apn1::nat apn1::neo MAT α*	This study
apn2Δ::APN2	*apn2::hyg APN2::neo MAT α*	This study

A

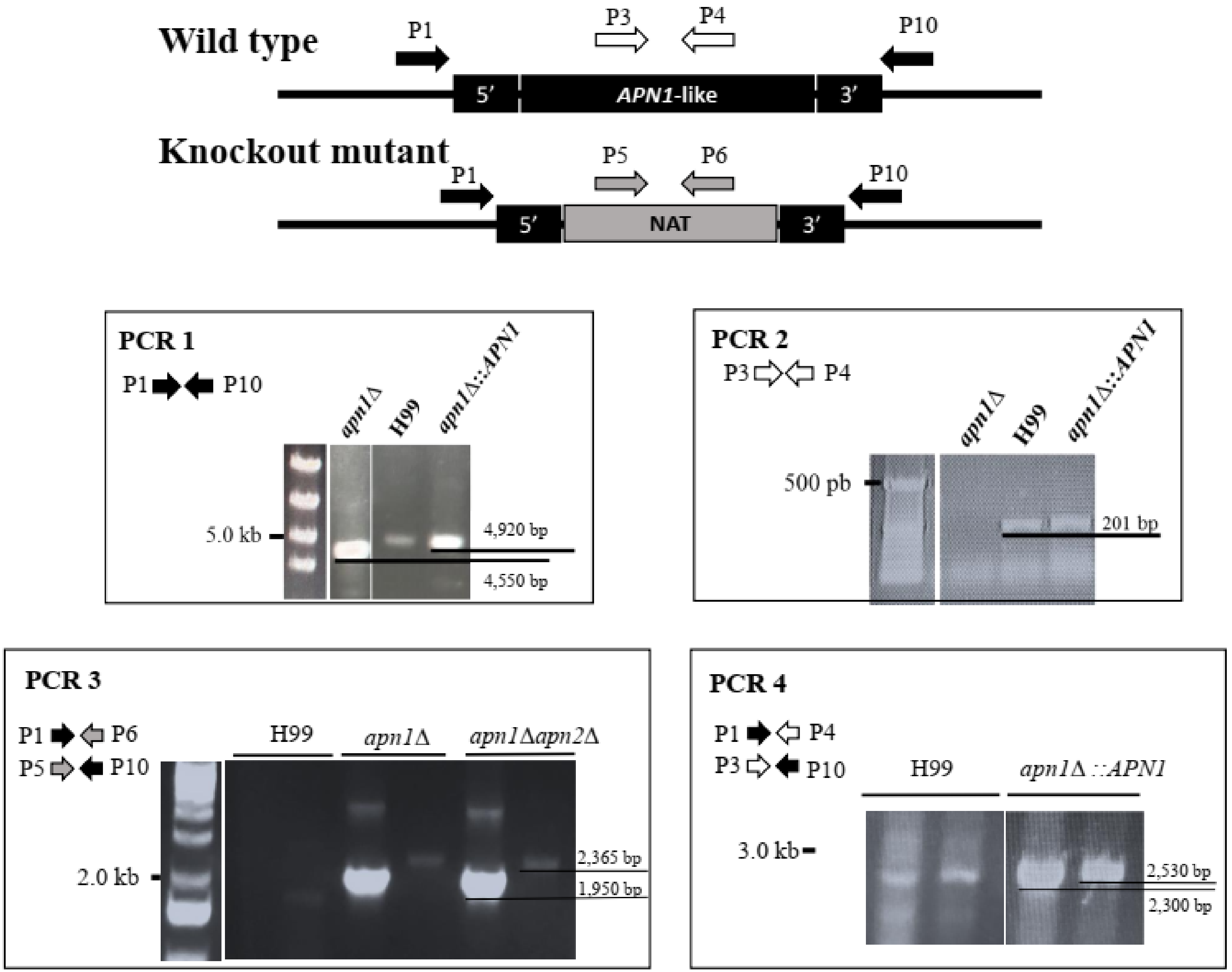

Wild type
P1
P3
P4
P10
5′
APN1-like
3′
Knockout mutant
P1
P5
P6
P10
5′
NAT
3′
PCR 1
P1 P10
apn1Δ
H99
apn1Δ::APN1
5.0 kb
4,920 bp
4,550 bp
PCR 2
P3 P4
apn1Δ
H99
apn1Δ::APN1
500 pb
201 bp
PCR 3
P1 P6
P5 P10
H99
apn1Δ
apn1Δapn2Δ
2.0 kb
2,365 bp
1,950 bp
PCR 4
P1 P4
P3 P10
H99
apn1Δ ::APN1
3.0 kb
2,530 bp
2,300 bp

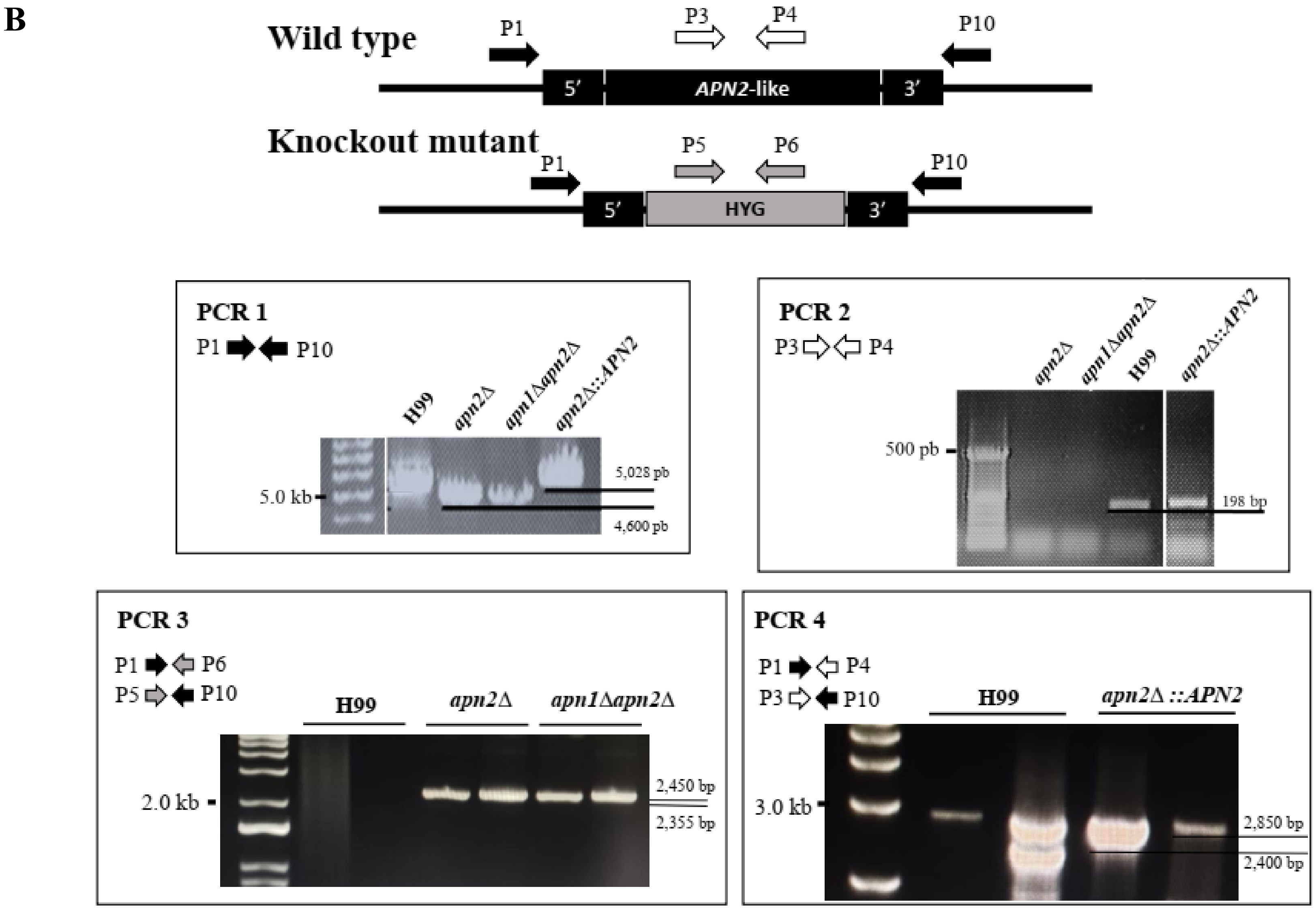

B
Wild type
P1
P3
P4
P10
5′
APN2-like
3′
Knockout mutant
P5
P6
HYG
PCR 1
P1
P10
H99
apn2Δ
apn1Δapn2Δ
apn2Δ::APN2
5.0 kb
5,028 pb
4,600 pb
PCR 2
P3
P4
apn2Δ
apn1Δapn2Δ
H99
apn2Δ::APN2
500 pb
198 bp
PCR 3
P1
P6
P5
P10
H99
apn2Δ
apn1Δapn2Δ
2.0 kb
2,450 bp
2,355 bp
PCR 4
P1
P4
P3
P10
H99
apn2Δ ::APN2
3.0 kb
2,850 bp
2,400 bp

Figure S1. Confirmation of knockout mutants by genomic DNA PCR amplification. Schematic representation of four PCR sets to confirm gene disruption. PCR 1 was performed with primers designed outside the 5′ upstream flaking region (5′) and the 3′ downstream flanking region (3′) (black arrows). Knockout mutants were expected to generate a single fragment in PCR1 that differed in size from that of the WT and specific reconstituted strains. PCR 2 consisted of multiplex reactions using specific primers for the target gene (white arrows). The target gene was expected to be amplified from the WT gDNA, whereas no amplicon was expected from the knockout mutants' DNA. PCR3 was performed with primers designed outside the 5′ and the 3′ regions (black arrows), and for the antibiotic resistance marker (HYG: Hygromicin B; NAT: Nourseotricin; Gray arrows). Amplicons in PCR 3 were expected to be generated only from the knockout mutants' DNA. PCR 4 was performed using a combination of flaking region primers and specific primers for the target gene (white arrows). In PCR4, the target genes were expected to be amplified only from the reconstituted strains DNA. **(A)** Electrophoretic analysis in 0.8% agarose gel stained with 0.5 μg/mL EtBr of *APN1*-like gene knockout and reconstituted strains' amplicons. **(B)** Electrophoretic analysis in 0.8% agarose gel stained with 0.5 μg/mL EtBr of *APN2*-like gene knockout and reconstituted strains' amplicons. The amplicons' size is indicated in base pairs (bp).

A

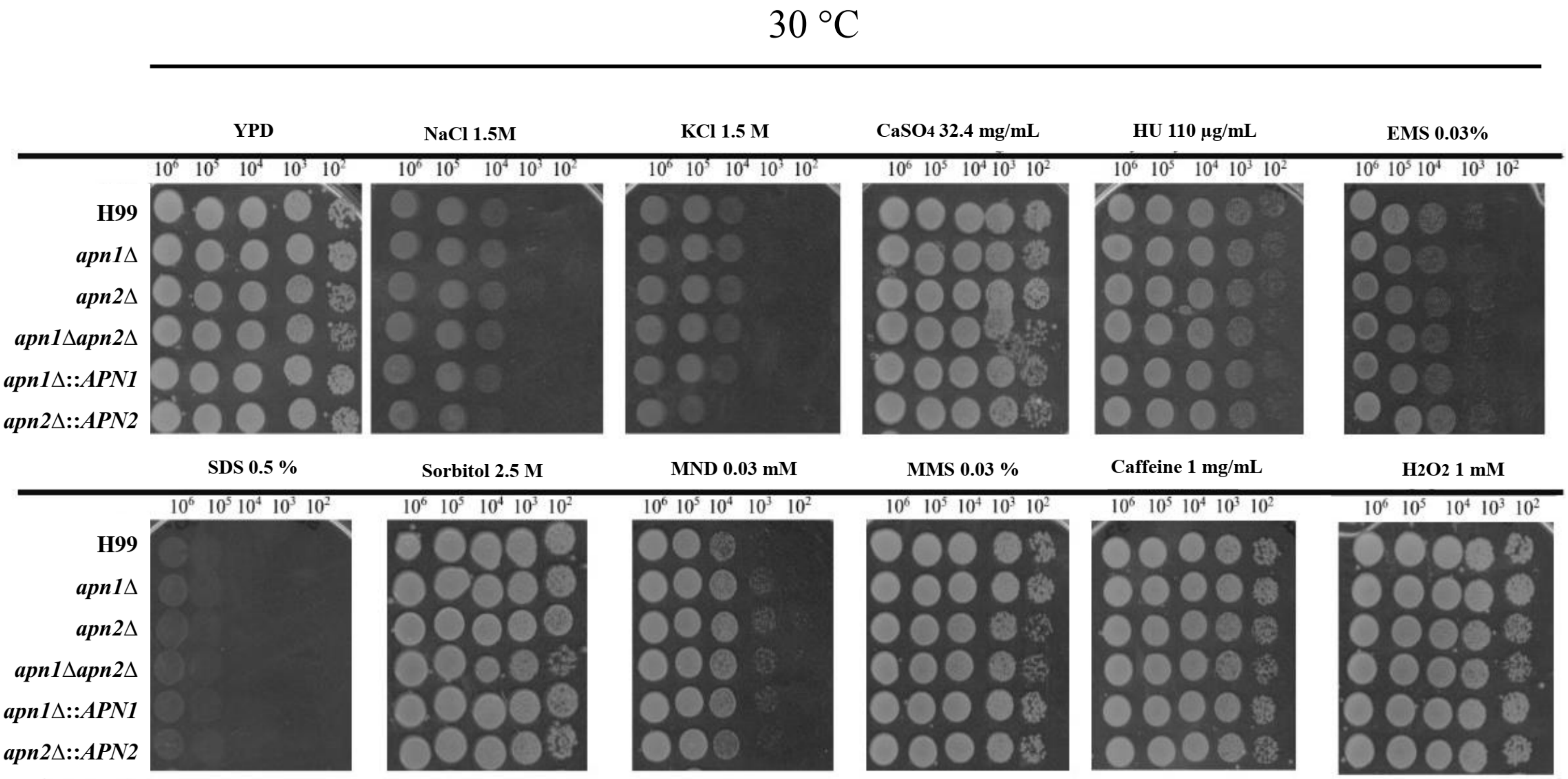
30 °C
YPD
NaCl 1.5M
KCl 1.5 M
CaSO4 32.4 mg/mL
HU 110 µg/mL
EMS 0.03%
SDS 0.5 %
Sorbitol 2.5 M
MND 0.03 mM
MMS 0.03 %
Caffeine 1 mg/mL
H2O2 1 mM
10^6 10^5 10^4 10^3 10^2
H99
apn1Δ
apn2Δ
apn1Δapn2Δ
apn1Δ::APN1
apn2Δ::APN2

B

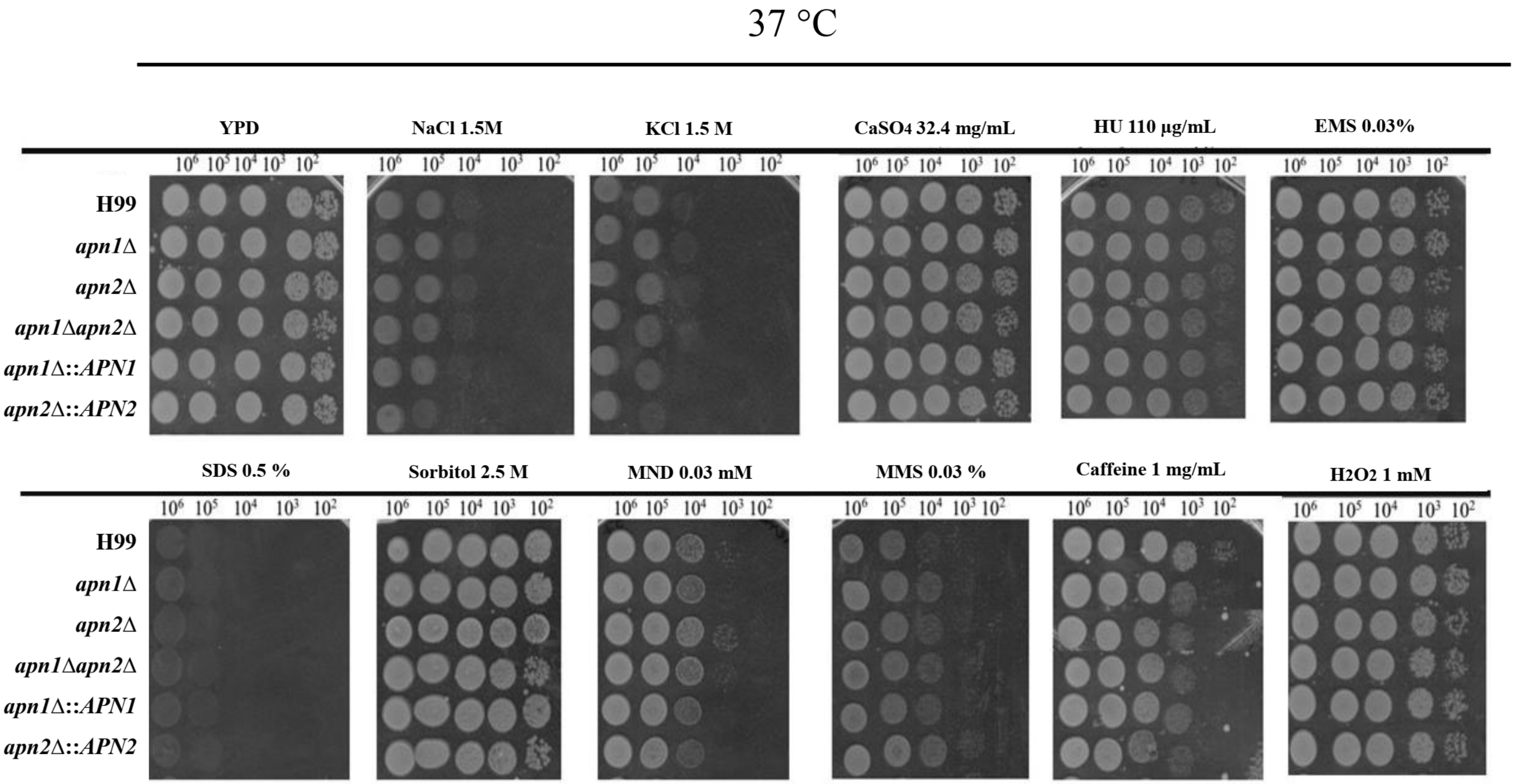

Figure S2. Temperature and chemical cell stress-related phenotypes of the *C. neoformans APN*-like genes' mutants. Ten-fold serial dilutions for the indicated strains were

spotted onto solid YPD supplemented with different compounds at 30 **(A)** and 37 °C **(B).** Growth was assessed after 48 h of incubation. EMS: ethylmethane sulfonate; MMS: methylethane sulfonate; HU: hydroxyurea; MND: Menadione).

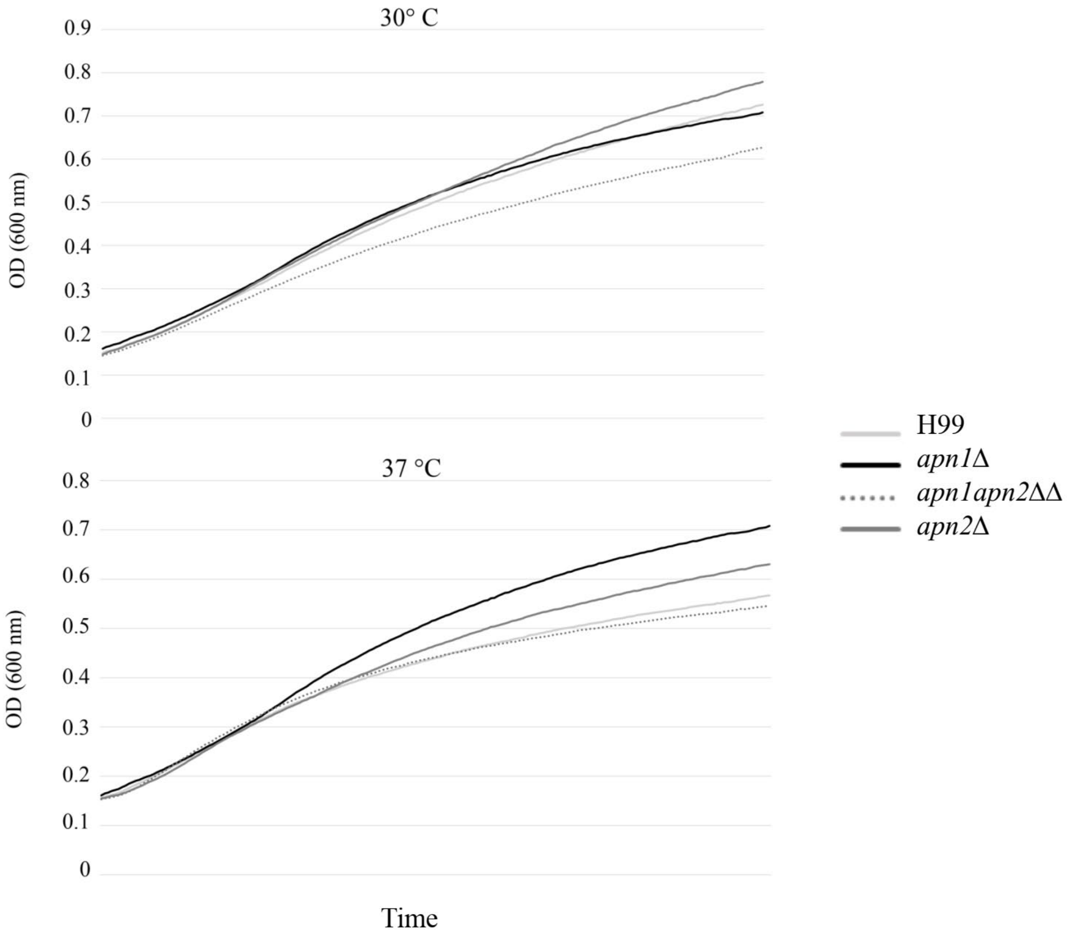

Figure S3. Growth curve of *C. neoformans* WT and mutant strains. Cells were grown overnight in liquid YPD and diluted to new cultures at 10^4 cells/mL per well. The cultures were then incubated at 30 or 37 °C in an automated microbiology growth curve analysis system (BioTek Eon Microplate Spectrophotometers, Winooski, VT, United States) and the absorbance (600 nm) was read every 30 min for 96 h. OD: optical density. The graphs represent the averages of three readings.

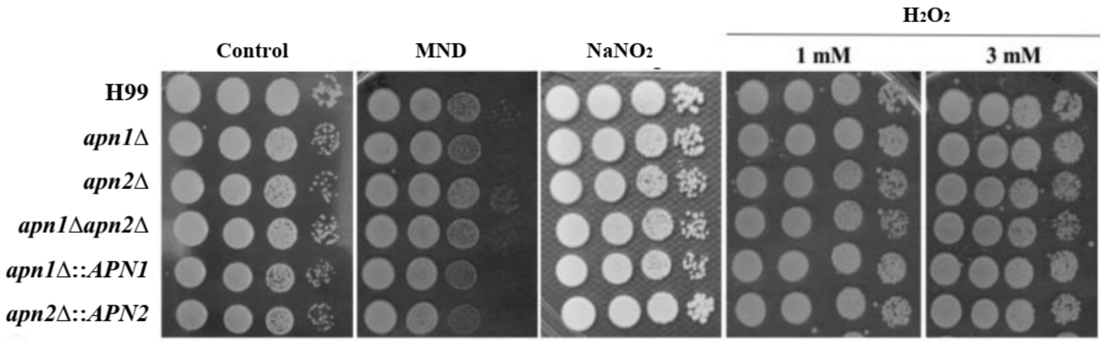

Figure S4. The ***apn1Δ*, *apn2Δ* and *apn1Δapn2Δ* mutants are not susceptible to oxidizing agents.** *C. neoformans* WT, mutant and reconstituted strains of yeasts were ten-fold serially diluted and spotted onto YPD agar supplemented with, 0.03 mM menadione (MND), 4 mg/mL sodium nitrite ($NaNO_2$) or 1 or 3 mM hydrogen peroxide (H_2O_2). Growth was accessed after 48 h of incubation, at 30 °C, protected from light. The results are representative of at least three independent experiments.

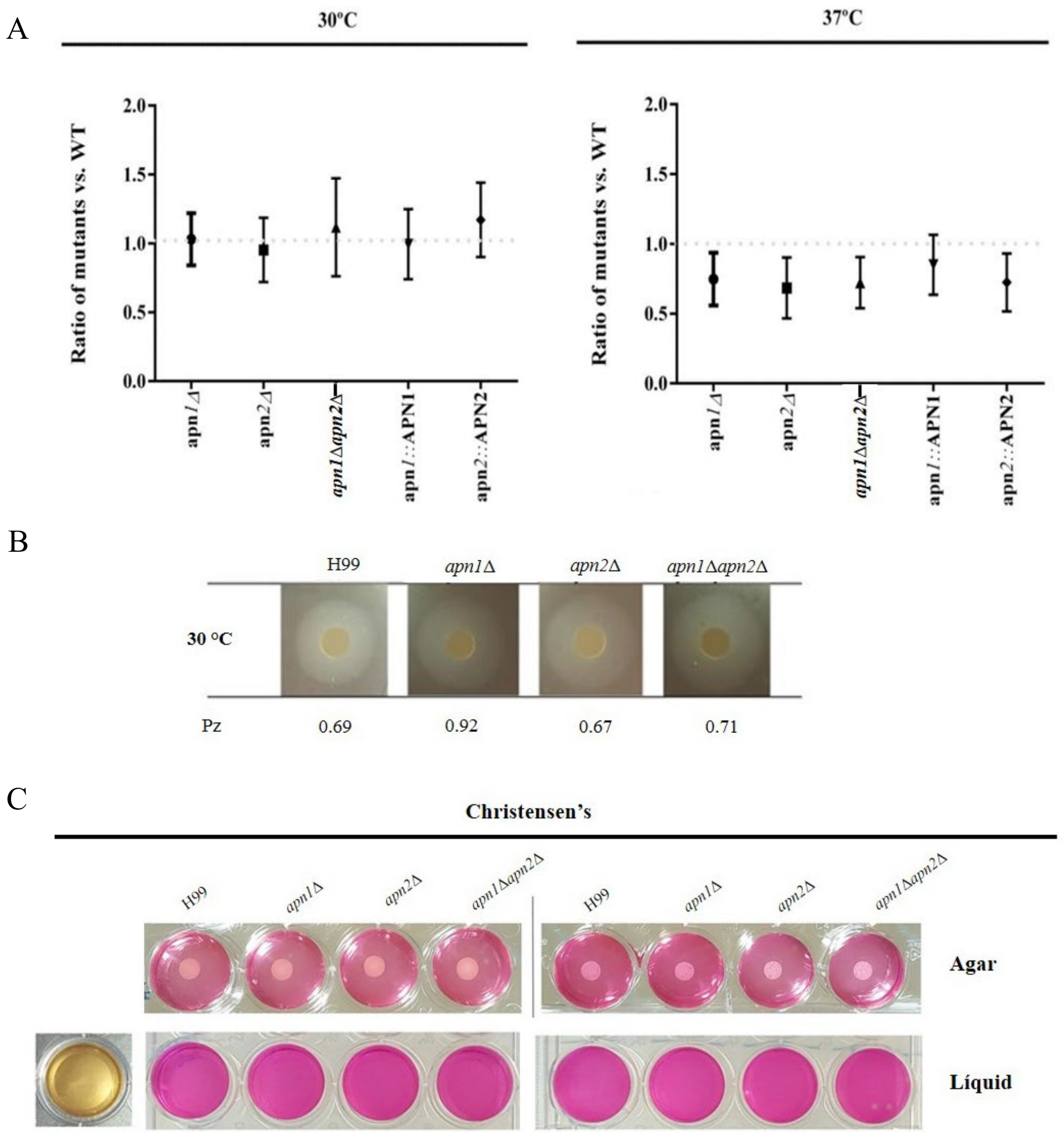

Figure S5. The capsule expansion, phospholipase and urease activities were unaffected by the disruption of Apn-like genes. **(A)** Determination of the capsule size. The strains were inoculated in minimum media (MM) at 30 or 37 °C for 72 h to induce capsule formation. Capsule size was visualized by India ink counterstaining and quantified by assessing the packed cell volume of normalized cell suspensions, expressed as a ratio to the WT strain. Data points represent averages of triplicate samples (+/− standard error). Statistical test: One-way ANOVA with Dunnett's post-test **(B)** Phospholipase activity assay. The yeast cells were spotted on Agar Egg Emulsion medium and incubated at 30 °C for 72 h. Hydrolysis halo sizes were measured according to the Price et al., (1982) protocol[84]. The phospholipase activity (Pz) was measured by the ratio of the colony diameter and the colony plus precipitation zone diameter after 72 h of growth at 30 °C. **(C)** Urease activity assay. Strains were cultivated for 48 h at 30 or 37 °C in liquid and in solid Christensen's urea medium. The shift from yellow to pink in the medium color is indicative of urease activity.

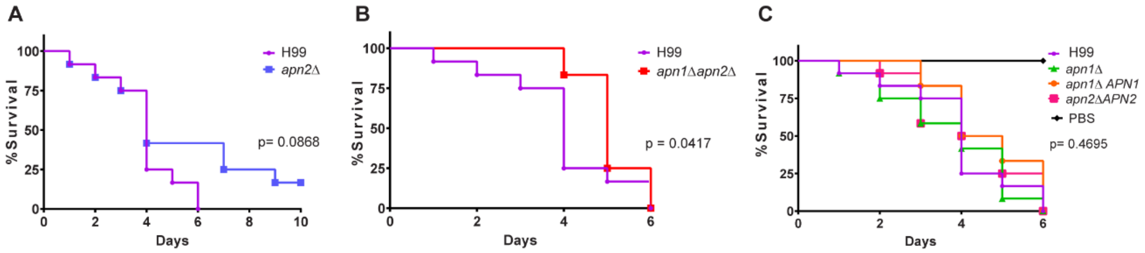

Figure S6. Evaluation of virulence of *C. neoformans APN* mutants using *G. mellonella* infection model. Larvae were infected in the last left proleg with 10 µL of 10^6 cell suspension of the indicated *C. neoformans* strains (WT, *apn1*Δ, *apn2*Δ, *apn1*Δ*apn2*Δ mutants) or 10 µL of PBS (mock control). After infection, they were incubated at 37 °C and daily monitored for survival. **(A)** Comparison between survival curves of larvae infected with H99 and *apn2Δ*. **(B)** Comparison between survival curves of larvae infected H99 and *apn1*Δ*apn2*Δ mutant. C) Survival curves of the larvae infected with WT, *apn1*Δ, *apn2*Δ, *apn1*Δ*apn2*Δ and the PBS control. Survival curves were analyzed using Log-rank (Mantel-Cox) test.

References

1. Perfect J.R., Lang S.D., Durack D.T. Chronic cryptococcal meningitis: a new experimental model in rabbits. *Am J Pathol.* 1980;101:177–194.
